# Structure of the atypical bacteriocin pectocin M2 implies a novel mechanism of protein uptake

**DOI:** 10.1111/mmi.12655

**Published:** 2014-06-18

**Authors:** Rhys Grinter, Inokentijs Josts, Kornelius Zeth, Aleksander W Roszak, Laura C McCaughey, Richard J Cogdell, Joel J Milner, Sharon M Kelly, Olwyn Byron, Daniel Walker

**Affiliations:** 1 Institute of Infection, Immunity and Inflammation, University of GlasgowGlasgow, G12 8QQ, UK; 2 Institute of Molecular Cell and Systems Biology, University of GlasgowGlasgow, G12 8QQ, UK; 3 School of Life Sciences, College of Medical, Veterinary and Life Sciences, University of GlasgowGlasgow, G12 8QQ, UK; 4 WestCHEM, School of Chemistry, College of Science and Engineering, University of GlasgowGlasgow, G12 8QQ, UK; 5 Unidad de Biofisica (CSIC-UPV/EHU)Barrio Sarriena s/n, 48940, Leioa, Vizcaya, Spain; 6 IKERBASQUE, Basque Foundation for ScienceBilbao, Spain

## Abstract

The colicin-like bacteriocins are potent protein antibiotics that have evolved to efficiently cross the outer membrane of Gram-negative bacteria by parasitizing nutrient uptake systems. We have structurally characterized the colicin M-like bacteriocin, pectocin M2, which is active against strains of *Pectobacterium* spp. This unusual bacteriocin lacks the intrinsically unstructured translocation domain that usually mediates translocation of these bacteriocins across the outer membrane, containing only a single globular ferredoxin domain connected to its cytotoxic domain by a flexible α-helix, which allows it to adopt two distinct conformations in solution. The ferredoxin domain of pectocin M2 is homologous to plant ferredoxins and allows pectocin M2 to parasitize a system utilized by *Pectobacterium* to obtain iron during infection of plants. Furthermore, we identify a novel ferredoxin-containing bacteriocin pectocin P, which possesses a cytotoxic domain homologous to lysozyme, illustrating that the ferredoxin domain acts as a generic delivery module for cytotoxic domains in *Pectobacterium*.

## Introduction

It is a dogma of colicin biology that after binding tightly to their cognate outer membrane (OM) receptor, colicins utilize an intrinsically unstructured translocation domain (IUTD) to recruit the inner membrane-bound Tol or Ton complex (Kleanthous, 2010; Housden *et al*., 2013). These complexes, which are responsive to the proton motive force (pmf), mediate translocation of the bacteriocin across the OM (Cascales *et al*., 2007; Housden *et al*., 2010). The formation of a colicin translocon has recently been visualized directly for the DNase-type colicin E9 through the isolation and imaging of the colicin in complex with its primary receptor BtuB, the trimeric porin OmpF, which allows passage of the IUTD across the OM and the periplasmic protein TolB, which is a component of the cell envelope-spanning TolABQR-Pal complex (Housden *et al*., 2013). Similarly, the TonB-dependent pore-forming colicin, colicin IA, uses one copy of the TonB-dependent receptor Cir as its primary receptor and a second copy as a translocation pathway for its IUTD to cross the OM to deliver a TonB-binding epitope to the periplasm (Jakes and Finkelstein, 2010). In addition to the colicins, which show a potent narrow spectrum of killing activity against strains of *E. coli* and other closely related bacteria, other colicin-like bacteriocins have also been characterized. These include the S-type pyocins from *Pseudomonas aeruginos*a, klebicins from *Klebsiella pneumonia* and syringacin M from *P. syringae* (Riley *et al*., 2001; Michel-Briand and Baysse, 2002; Barreteau *et al*., 2009). The recently determined structures of the M-class bacteriocins pyocin M and syringacin M showed that like colicin M, these bacteriocins possess a 30- to 40-amino-acid IUTD, which is essential for translocation, indicating that translocation across the OM likely occurs through the same mechanism as the colicins (Zeth *et al*., 2008; Barreteau *et al*., 2012a,b[Bibr b4],[Bibr b5]; Grinter *et al*., 2012b).

We recently described the novel M-class bacteriocins pectocin M1 and M2, which are produced by and active against strains of the soft-rot phytopathogens *Pectobacterium atrosepticum (Pba)* and *Pectobacterium carotovorum (Pbc)* (Grinter *et al*., 2012a; 2013[Bibr b19],[Bibr b21]). The domain structure of these proteins suggested that they challenge the dogma that an IUTD is the universal mechanism by which colicin-like bacteriocins achieve translocation. Pectocin M1 and M2 consist of an M-class cytotoxic domain with lipid II degrading activity, fused to a plant-like ferredoxin domain (Grinter *et al*., 2012a; 2013[Bibr b19],[Bibr b21]). This ferredoxin domain, which contains an intact [2Fe-2S] iron–sulphur cluster, substitutes for the helical receptor binding domain and IUTD of the M-class bacteriocins discussed above, that are required to deliver the cytotoxic domain to the periplasm. Further to this, we observed that the addition of plant ferredoxin to strains of *Pba* and *Pbc* exposed to the pectocins inhibited bacteriocin-induced killing (Grinter *et al*., 2012a). These observations show first, that *Pba* and *Pbc* possess an OM receptor able to bind ferredoxin and second, that pectocins M1 and M2 parasitize this receptor to target and ultimately gain entry to susceptible cells. The role of ferredoxin binding for these plant pathogens is apparent under iron-limiting conditions where, in the presence of plant ferredoxin, some strains of *Pectobacterium* spp. show strongly enhanced growth (Grinter *et al*., 2012a). This effect is not observed on addition of the mammalian ferredoxin homologue, adrenodoxin, which also contains a [2Fe-2S] iron–sulphur cluster (even at greatly increased concentrations), indicating a high level of specificity for plant ferredoxin. Similarly, adrenodoxin is not able to rescue cells from pectocin M-induced killing (Grinter *et al*., 2012a). Thus, like other colicin-like bacteriocins, pectocins M1 and M2 parasitize an existing nutrient uptake system to gain entry into target cells. However, for these bacteriocins the mechanism is overt and unprecedented, with the direct utilization of ferredoxin, a protein from which *Pectobacterium* spp. is able to directly acquire iron, as the targeting region of the bacteriocin (Grinter *et al*., 2013).

In order to gain further insight into the mechanism through which pectocins M1 and M2 gain entry into target cells, we have used X-ray crystallography and small angle X-ray scattering along with *in silico* modelling approaches to characterize the structural and dynamic properties of pectocin M2. Our data show that there is a high degree of conformational flexibility between the ferredoxin and colicin M-like cytotoxic domain through movement of a linking helix and definitively show that the protein lacks the flexible IUTD that is characteristic of all other characterized colicin-like bacteriocins. The lack of an IUTD indicates that the ferredoxin-containing pectocins utilize an existing ferredoxin uptake mechanism to cross the OM, without direct interaction with the Tol or Ton complexes in the periplasm. Additionally, we have determined the existence of an additional ferredoxin-containing bacteriocin, pectocin P, which possesses a cytotoxic domain that is a structural homologue of lysozyme, illustrating that ferredoxin can act as a generic module for the delivery of structurally diverse cytotoxic proteins to the periplasm.

## Results

### The crystal structure of pectocin M2

**Figure 1:**
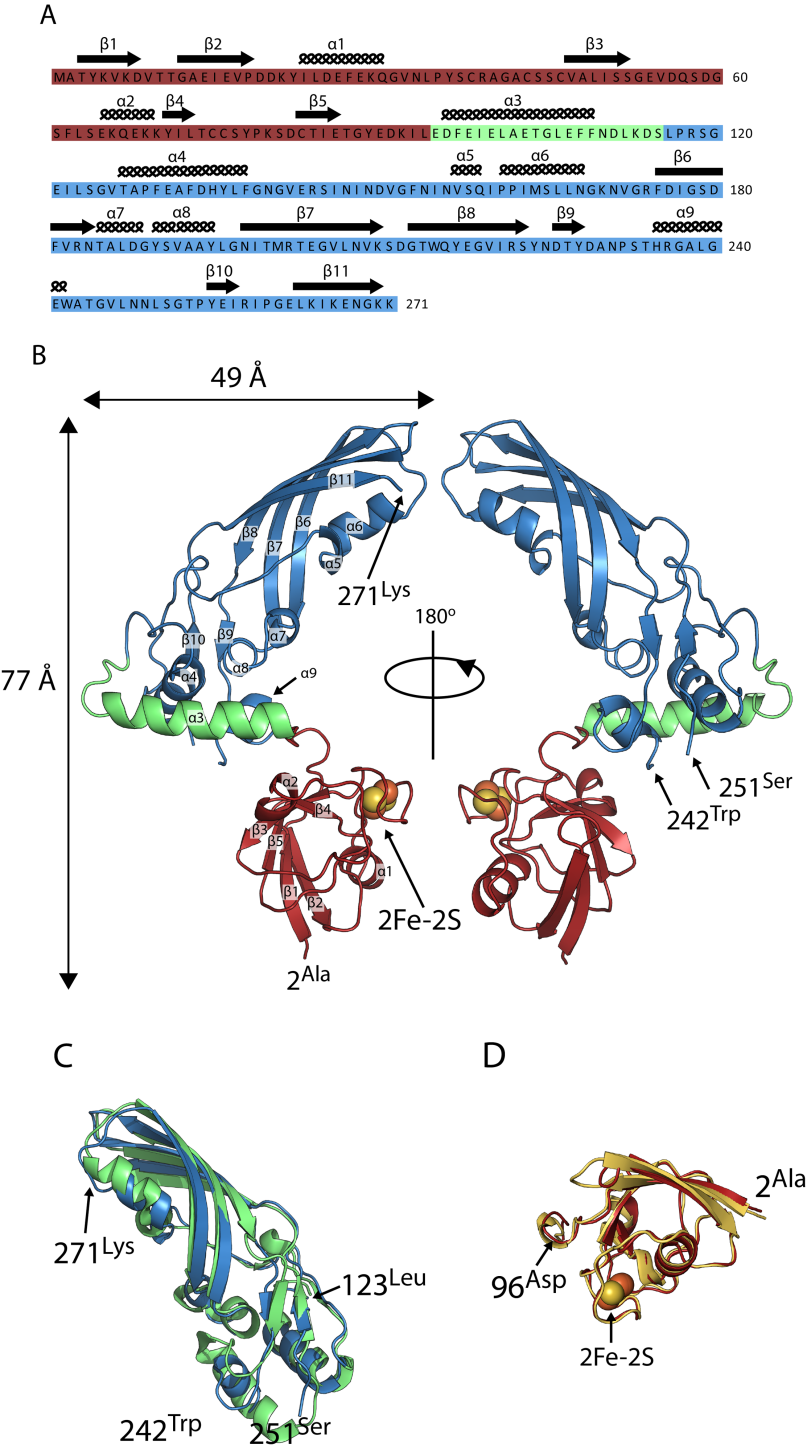
The crystal structure of pectocin M1 reveals a ferredoxin-containing colicin-like bacteriocin that lacks an IUTD.A. Amino acid sequence of pectocin M2, showing structural domains (ferredoxin domain = red, linker helix = green, catalytic domain = blue) and annotated with secondary structure.B. Schematic of the crystal structure of pectocin M2 observed in the *P*2_1_ crystal form, with the cytotoxic domain in blue, plant-like ferredoxin domain in red and linker helix in green. The [2Fe-2S] iron–sulphur cluster is represented by spheres.C. Schematic of cytotoxic domain of pectocin M2 aligned with that of colicin M (PDB ID = 2XMX) (backbone r.m.s.d. = 1.65 Å, pectocin M2 residues = 123–271, colicin M residues = 123–271).D. Schematic of the ferredoxin domain of pectocin M2 aligned with that of spinach ferredoxin (PDB ID = 1A70) (backbone r.m.s.d. = 0.6 Å, pectocin M2 residues = 2–96, spinach ferredoxin residues 2–96).

**Figure 2:**
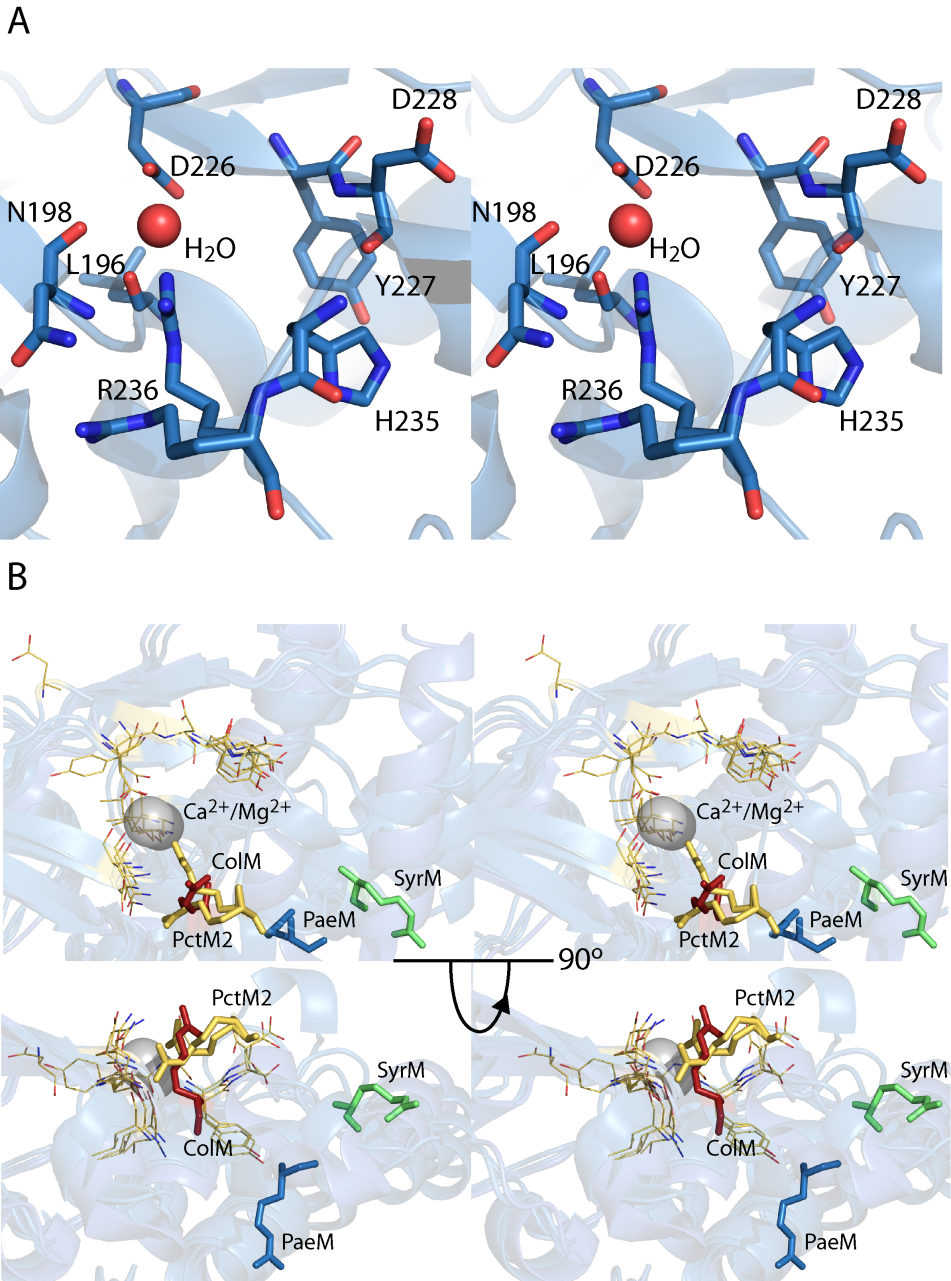
Colicin M-class bacteriocins possess a highly flexible active site.A. A stereo view of a stick model of the key active site residues of pectocin M2, showing a water molecule occupying the key metal binding site of the enzyme.B. A stereo view of the overlay of the catalytic site from all structurally characterized colicin-M class bacteriocins, showing conformational variability of the key catalytic arginine. Key arginine shown as sticks and colour coded according to structure; green = syringacin M (PDB ID = 4FZL), blue = pyocin M (PDB ID = 4G75), red = colicin M (PDB ID = 2XMX) and yellow = pectocin M2 (PDB ID = 4N58), All other catalytically important residues shown as lines in yellow.

In initial crystallization trials for pectocin M2, characteristic red-brown crystals of this ferredoxin-containing bacteriocin formed with PEG 3350 and ammonium sulphate as the precipitants. Data from these crystals were collected to 2.3 Å in the space group *P*2_1_ and phased using anomalous scattering data from the metal centres of the [2Fe-2S] iron–sulphur cluster. The structure of pectocin M2 revealed an N-terminal domain with the predicted ferredoxin-fold (residues 2–94, in red), separated from the colicin M-like cytotoxic domain (residues 116–271, in blue) by a linker region (residues 95–115, in green) that forms an α-helix (Fig. 1A and B). There is a significant difference in the orientation of the cytotoxic and ferredoxin domains of the two pectocin M2 molecules in the asymmetric unit (ASU) with a root mean square deviation (r.m.s.d.) of 3.4 Å, between main-chain atoms (Fig. S1). The fold of the pectocin M2 ferredoxin domain is identical (r.m.s.d. 0.60 Å) to that of spinach ferredoxin (PDB ID = 1A70) and the C-terminal cytotoxic domain is highly similar to the lipid II-cleaving catalytic domains of colicin M (PDB ID = 2XMX, r.m.s.d. 1.7 Å) (Fig. 1C and D) (Zeth *et al*., 2008). The crystal structure of pectocin M2 adds to a growing body of structural and biochemical data on colicin M-like cytotoxic domains (Zeth *et al*., 2008; Barreteau *et al*., 2010; 2012b; Helbig and Braun, 2011; Grinter *et al*., 2012b). We confirmed the enzymatic activity of pectocin M1 and M2 by a lipid II hydrolysis assay (Fig. S2). In the recently solved structures of pyocin M (PaeM) and syringacin M a divalent metal ion (Ca^2+^ or Mg^2+^) is co-ordinated by a key catalytic aspartic acid side-chain in conjunction with two backbone carbonyls. Mg^2+^, Ca^2+^ or Mn^2+^ ions are required for catalytic activity of M-class bacteriocins, and analysis of these proteins has shown co-ordination at this position to be essential for activity (Grinter *et al*., 2012b). In the pectocin M2 structure this key aspartate (D226) adopts an analogous conformation. However, no density for a metal ion is observed in this position, which is occupied by a water molecule (Fig. 2A). The absence of a metal ion is unsurprising given the lack of divalent ions and the high ionic strength of the crystallization conditions.

Comparative analysis of the catalytic domains of colicin M homologues reveals significant variation between the structures. In the structures of pyocin M and syringacin M a key conserved arginine is located distant from other conserved residues creating an open active-site cleft. In contrast, in the pectocin M2 structure this residue (R236) is orientated towards the other key catalytic residues (Fig. 2B). The electron density for R236 permitted modelling of two conformations, one within hydrogen bonding distance of the aspartic acid co-ordinated water and the other forming a hydrogen bond with N184. In this conformation, R236 creates a defined active site tunnel which would enable co-ordination of the lipid II and positioning of the pyrophosphate group in close proximity to all key catalytic residues (Fig. 2A and B).

In contrast to the compact structures of the homologous bacteriocins, colicin M, pyocin M and syringacin M, where the receptor binding and catalytic domains are not separated by linker regions and do not form obviously structurally distinct elements (Zeth *et al*., 2008; Barreteau *et al*., 2012b; Grinter *et al*., 2012b), the catalytic and receptor binding domains of pectocin M2 do not form extensive interactions. The relative orientation of the ferredoxin domain, linker region and cytotoxic domain gives rise to a non-linear dog-leg structure. Interestingly, and again in contrast to colicin M, pyocin M and syringacin M, the N-terminal region of pectocin M2 lacks a disordered or flexible IUTD that is otherwise characteristic of the colicin-like bacteriocins, with the entire N-terminus being integral to the globular ferredoxin domain. These data suggest a mechanism of uptake distinct from closely related colicin-like bacteriocins.

### Pectocin M2 is flexible

Given that pectocin M2 lacks an IUTD required to contact the Tol or Ton complexes in the periplasm and mediate translocation of this protein across the outer membrane, alternative mechanisms of uptake must be considered. One possibility is that the entire bacteriocin passes through the lumen of its OM receptor. Since proteins involved in iron uptake are invariably TonB-dependent receptors that possess large 22-stranded β-barrels this may be plausible. However, such a mechanism would only be feasible if pectocin M2 were flexible and significant rearrangement of the dog-leg configuration observed in the crystal structure could be achieved. The observation that there is a relatively large difference in orientation between the cytotoxic and ferredoxin domains in the monomers of the ASU (Fig. S1) is suggestive of such flexibility and indicates that the crystal structure may not be wholly representative of pectocin M2 in solution.

**Figure 3:**
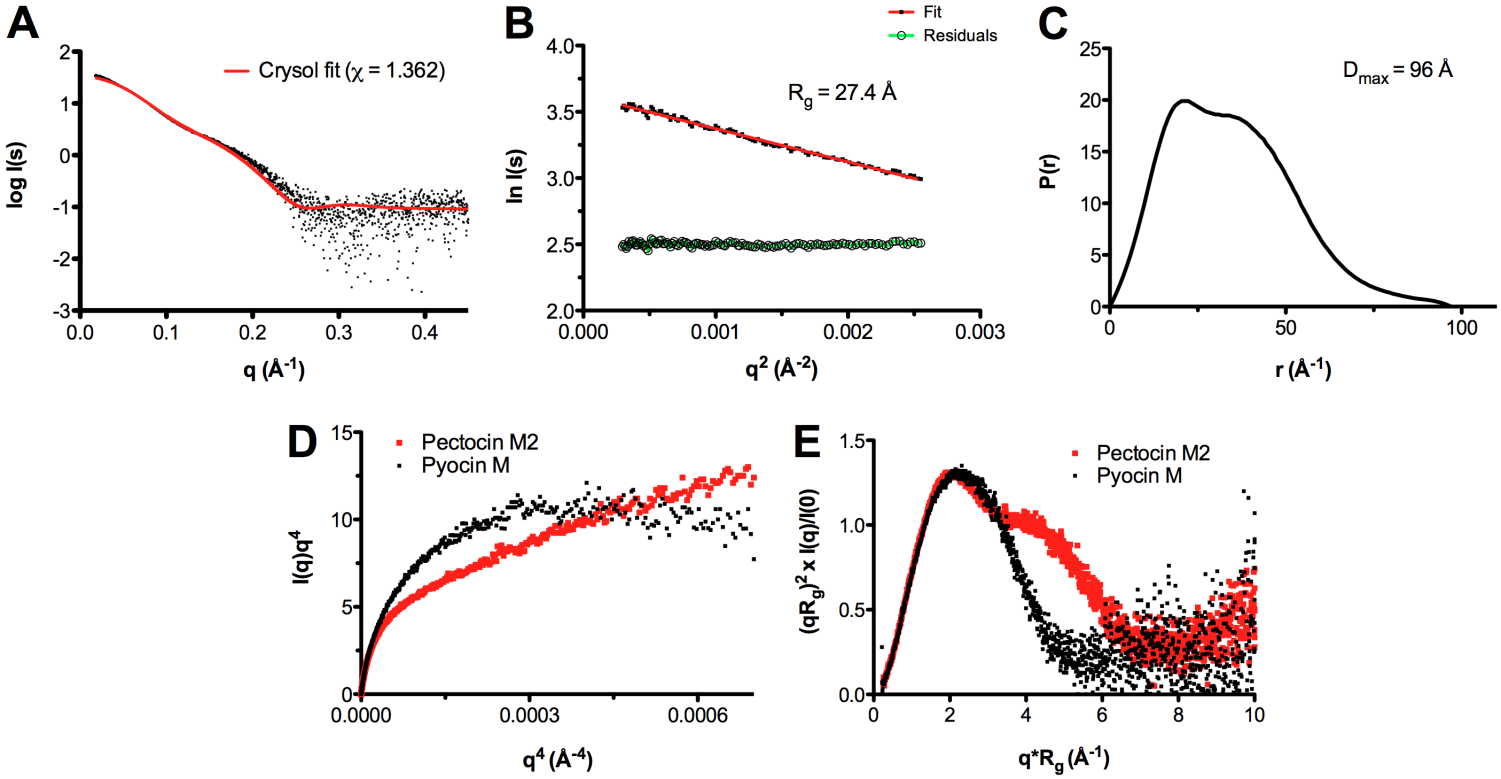
SAXS shows pectocin M2 is flexible.A. Overlay of the experimentally determined pectocin M2 SAXS curve (black points) with the scattering curve computed with CRYSOL from the *P*2_1_ crystal structure (red line) produces a fit (χ = 1.362) with visible deviations between the data, especially evident at low angles, suggesting that the crystal structure is more compact than that of pectocin M2 in solution.B. Derivation of *R*_g_ from a Guinier analysis (red) of the scattering curve; residuals of the fit are in green.C. Pair-distance distribution plot from experimental scattering data for pectocin M2 exhibiting two maxima which highlights the bimodal character of the molecule in solution. The *D*_max_ of the particle is 96 Å.D. Porod-Debye and (E) normalized Kratky plots for pectocin M2 imply increased flexibility of the protein in solution (red). Pyocin M (black), a protein of similar molecular weight with a relatively rigid structure and strong inter-domain contacts is used as a control.

To assess the conformational flexibility of pectocin M2 we performed small angle X-ray scattering (SAXS). SAXS data were obtained for a range of pectocin M2 concentrations. Comparison of these data with a theoretical scattering curve generated, using CRYSOL (Svergun *et al*., 1995), from the pectocin M2 crystal structure shows there are obvious differences between the theoretical curve and experimental scattering data (Fig. 3A). In addition, the radius of gyration (*R*_g_ = 27 Å) obtained from Guinier analysis of the experimental scattering data is somewhat larger than that calculated from the pectocin M2 crystal structure (*R*_g_ = 24 Å) using SOMO (Rai *et al*., 2005) (Fig. 3B). Consistent with this, the p(r) function, which describes the paired set of vectors between all the electrons within the protein, indicates a maximum particle size (*D*_max_ = 96 Å, Fig. 3C) that is much greater than the maximum dimension of the pectocin M2 crystal structure (77 Å, Fig. 1B). These data suggest that the pectocin M2 crystal structure is not wholly representative of the conformational ensemble present in solution and that this protein adopts an elongated conformation, implying inter-domain flexibility.

To test this idea further, we examined the Porod-Debye plot for pectocin M2, where scattering decay is examined as *I*(q)q^4^ as a function of q^4^. This analysis reports directly on particle flexibility and typically for compact globular particles an asymptotic plateau is reached for the low q part of the data. However, for pectocin M2 no discernible plateau was observed (Fig. 3D). For comparison, we also obtained scattering data for pyocin M which, as with colicin M and syringacin M, forms a compact structure and similarly analysed these data (Barreteau *et al*., 2012b). In contrast to the curve obtained for pectocin M2, the Porod-Debye plot for pyocin M reached a plateau confirming its rigidity and compactness (Fig. 3D). In addition, the Kratky plot [*I*(q)q^2^ versus q] for pectocin M2 normalized to the scattering intensity *I*(0) and *R*_g_, has two maxima with increasing scattering at higher angles. The Kratky plot reports directly on inter-domain flexibility and for pectocin M2 is consistent with a two-domain protein connected by a flexible linker (Fig. 3E). In comparison, there is a single maximum in the pyocin M Kratky plot, consistent with its single domain-like globular structure. Taken together these analyses indicate that pectocin M2 is flexible and adopts conformations distinct from that observed in the crystal structure.

### Pectocin M2 can adopt a highly extended conformation and exists as two distinct subpopulations in solution

**Figure 4:**
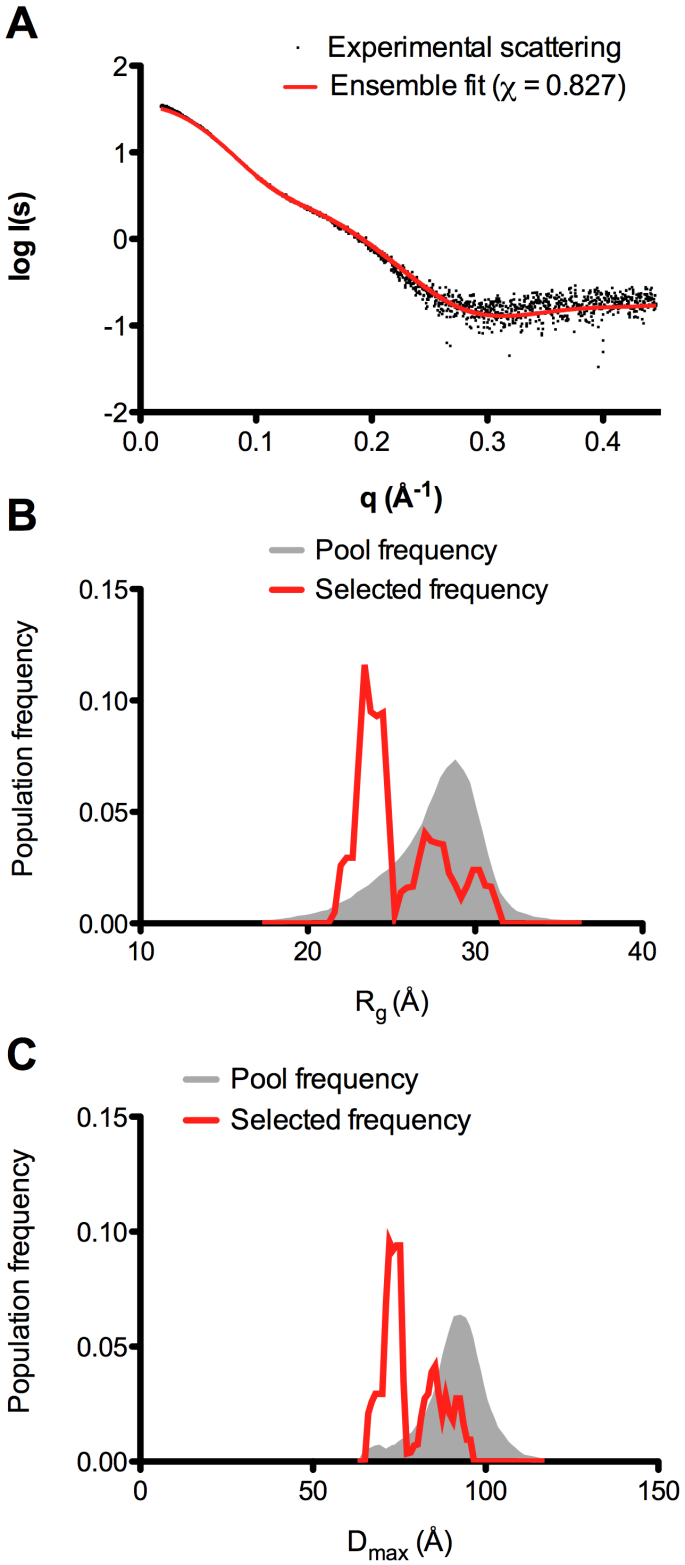
Analysis of conformational heterogeneity of pectocin M2 reveals compact and extended ensembles in solution.A. Overlay of scattering curves of pectocin M2 between experimental data and the best ensemble selected by GAJOE indicates improved fit to the scattering data (χ = 0.827).B and C. *R*_g_ (B) and *D*_max_ (C) distribution of solution ensembles selected by a genetic algorithm using GAJOE from a pool of 5000 random conformers of pectocin M2. Compact and elongated molecule were both selected implying that the protein is conformationally heterogeneous in solution allowing for significant inter-domain re-arrangements about the linker helix (residues 96–115).

To determine if the SAXS data for pectocin M2 could be better described by an ensemble of conformations we first used discrete molecular dynamics (DMD) simulations (Shirvanyants *et al*., 2012) to explore the accessible conformational states of pectocin M2 and generated a random pool of 5000 possible conformationals using the crystal structure of pectocin M2. Next, we used a genetic algorithm implemented in the program GAJOE (Petoukhov *et al*., 2012) to select for ensembles of these models that would better describe our SAXS data. Model selection was successful as judged by the close correlation of the theoretical scattering curve generated from the selected ensemble with the experimental SAXS data (Fig. 4A), indicating that our scattering data are best described by an ensemble of pectocin M2 conformers in solution. Interestingly, the selected ensembles show a bimodal distribution in comparison with the random pool of DMD-generated pectocin M2 models when the population frequency is plotted against *R*_g_ or *D*_max_ (Fig. 4B and C). Thus, in the population of selected conformations, we frequently find a compact conformation described by the first peak (with maxima at approximately 23 and 75 Å for *R*_g_ and *D*_max_ respectively) that approximates closely to the conformation found in the pectocin M2 *P*2_1_ crystal structure for which *R*_g_ and *D*_max_ were calculated as 24 and 77 Å respectively. The second peak represents an ensemble of pectocin M2 conformers in an extended conformation with *D*_max_ values ranging up to 98 Å, which correlates closely with the experimentally determined value of *D*_max_ (96 Å). These analyses suggest that pectocin M2 can adopt both bent and elongated linear conformations in solution.

**Figure 5:**
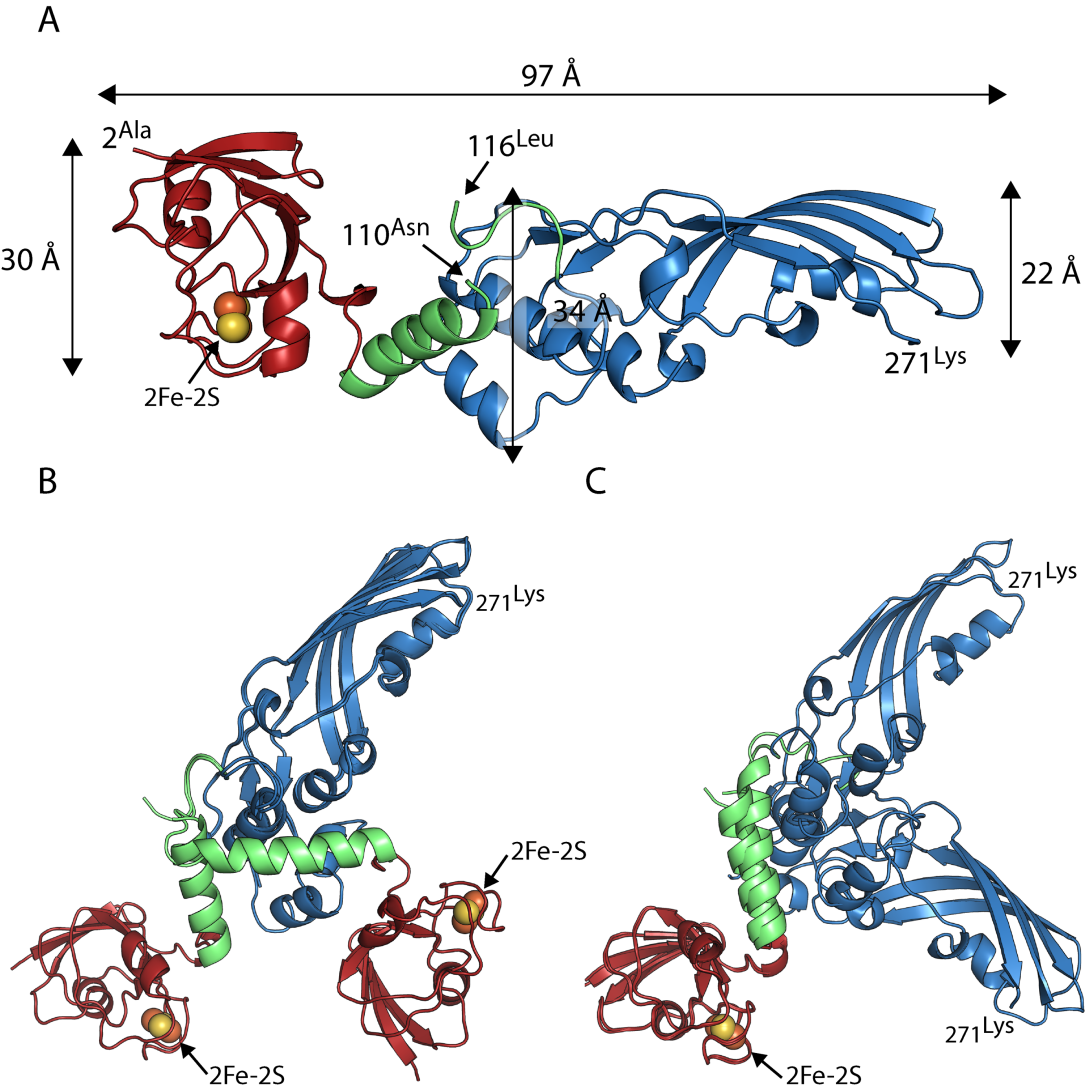
Pectocin M2 *P*3_1_21 structure confirms extended conformation predicted by solution scattering.A. Pectocin M2 in *P*3_1_21 crystal form is highly elongated, consistent with the extended conformation predicted from solution scattering and DMD simulations.B. Alignment of the catalytic domains of the *P*2_1_ and *P*3_1_21 crystal forms of pectocin M2, illustrating the difference in orientation between the ferredoxin and linker regions.C. Alignment of the ferredoxin domains of the *P*2_1_ and *P*3_1_21 crystal forms of pectocin M2, illustrating the difference in orientation of the catalytic domains.

The bimodal distribution of the selected ensembles suggests discrete populations in solution, the more compact of which is similar to the conformation observed in the *P*2_1_ crystals of pectocin M2. In an attempt to capture the more elongated conformation *in crystallo*, thus validating our solution scattering and modelling data, we repeated crystallization of pectocin M2. A custom re-crystallization screen was devised exploiting information from the initial trails. Crystals were obtained in a number of conditions from this screen and were tested for diffraction as well as space group and unit cell variation, which is indicative of novel packing. A form with the radically different space group of *P*3_1_21 was chosen for optimization, which yielded crystals diffracting to 1.86 Å. As an alternative domain arrangement to the *P*2_1_ form was expected, data from this crystal form were again phased using anomalous data from the metal centres of the [2Fe-2S] cluster. During model building from these data it was immediately apparent that in this crystal form pectocin M2 did indeed adopt an elongated conformation (Fig. 5A). The calculated *R*_g_ and *D*_max_ for this structure were 28 and 97 Å respectively. These values correlate well with the extended population from the DMD simulation, suggesting that this structure is representative of the second elongated pool indentified by our modelling. Alignment of this elongated (*P*3_1_21) form and the original *P*2_1_ form, based on their cytotoxic or ferredoxin domains, show a major difference in the relative orientations of these domains (Fig. 5B and C).

### The ferredoxin domain is a generic module for the delivery of cytotoxic domains to the periplasm

**Figure 6:**
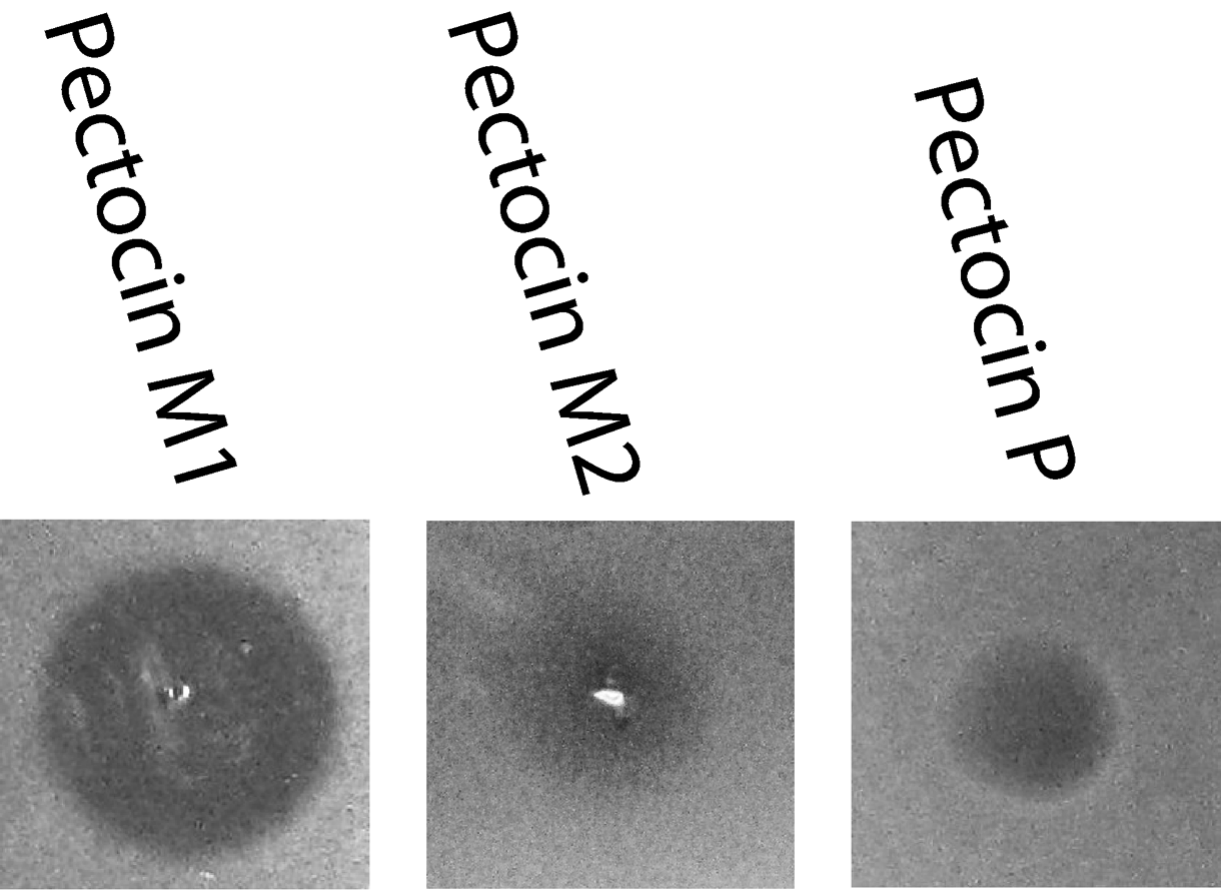
Activity of pectocin P against *Pectobacterium atrosepticum* LMG 2386. Purified pectocins M1, M2 and P (2 mg ml^−1^) were spotted onto a soft agar overlay seeded with *Pba* LMG 2386. Clear zones indicate inhibition of growth due to the cytotoxic effect of pectocins on cells.

In addition to pectocin M1 and M2, we previously identified a putative third member of the ferredoxin-containing bacteriocin family, designated pectocin P (Grinter *et al*., 2012a). The open reading frame for pectocin P, identified in the genome of *Pectobacterium carotovorum* subsp*. carotovorum* WPP14, consists of an N-terminal ferredoxin domain, connected to a pesticin-like cytotoxic domain, which is analogous to T4 lysozyme. Similar to pectocins M1 and M2, there is no sequence N-terminal of the ferredoxin domain, so this bacteriocin also lacks an IUTD. To confirm that this open-reading frame encodes an active bacteriocin, we tested the cytotoxic activity of recombinantly expressed and purified pectocin P against diverse *Pectobacterium* isolates. For this test we utilized a solid growth inhibition assay conducted in parallel with pectocins M1 and M2 (Fyfe *et al*., 1984). As with pectocins M1 and M2, limited inhibition of growth was observed under iron-replete conditions (LB agar). However, under iron-limiting conditions, inhibition of growth was observed for 17 of the 19 strains (Fig. 6, Table S1). The existence of pectocin P, an additional ferredoxin-containing bacteriocin with no N-terminal IUTD and a pesticin-like cytotoxic domain, provides strong supporting evidence that the ferredoxin domain acts as a generic module for the delivery of cytotoxic domains to the periplasm in *Pectobacterium* spp. The cytotoxic domains of both M-class bacteriocins and pesticin have been studied extensively and there is no indication that they possess any intrinsic capacity to cross the OM (Barreteau *et al*., 2010; Helbig and Braun, 2011; Lukacik *et al*., 2012; Patzer *et al*., 2012), indicating that ferredoxin uptake represents an unprecedented example of receptor-mediated protein uptake for nutrient acquisition in bacteria.

## Discussion

In this work we present the structure and solution properties of the atypical bacteriocin pectocin M2, which consists of a fusion between a colicin M-like cytotoxic domain and a plant-derived ferredoxin domain. We have previously demonstrated that *Pectobacterium* spp. are able to acquire iron directly from plant ferredoxin under iron-limiting conditions through a receptor mediated process and that the bacteriocins pectocin M1 and M2 parasitize this system for cell entry through presentation of a ferredoxin domain (Grinter *et al*., 2013). In this study we provide an insight into how this uptake occurs by showing that pectocin M2 has an unprecedented structure among bacteriocins in that it lacks an IUTD. Additionally, we definitively show that pectocin M2 is highly flexible in solution fluctuating between compact and extended conformations.

All iron-uptake systems identified to date in Gram-negative bacteria, either siderophore based or targeting a protein substrate, utilize a TonB-dependent receptor to transport iron across the outer membrane (Faraldo-Gomez and Sansom, 2003). Likewise, binding and parasitization of these receptors for cell entry is a characteristic trait of colicin M, and other characterized colicins and pyocins, including the E-type colicins (E1-E9), colicins A, B, D, IA and IB and pyocins S1-S5 (Loftus *et al*., 2006; Buchanan *et al*., 2007; Cascales *et al*., 2007; Denayer *et al*., 2007; Devanathan and Postle, 2007; Elfarash *et al*., 2012). As such, while it is yet to be confirmed that a TonB-dependent receptor is responsible for mediating ferredoxin iron and pectocin uptake, this class of protein is by far the most likely candidate.

In bacteriocins the IUTD normally functions to deliver an epitope to the periplasm, which mediates binding to the Tol or Ton complexes. This direct interaction occurs between the colicins and TolB for group A colicins and TonB for group B colicins and is essential for uptake of these bacteriocins. In addition to their subversion for bacteriocin import, Tol and Ton complexes have a general physiological role in the bacterial cell. The Ton complex provides the energy required for the import of iron containing siderophores and related substrates through TonB-dependent receptors, by interaction with the receptor plug domain subsequent to binding of the substrate on the outer surface of the receptor (Noinaj *et al*., 2010). Given the universal role of TonB-dependent receptors in iron transport across the outer membrane and their parasitization by colicin-like bacteriocins, it is reasonable to speculate that the ferredoxin/pectocin receptor is a member of this class of protein.

**Figure 7:**
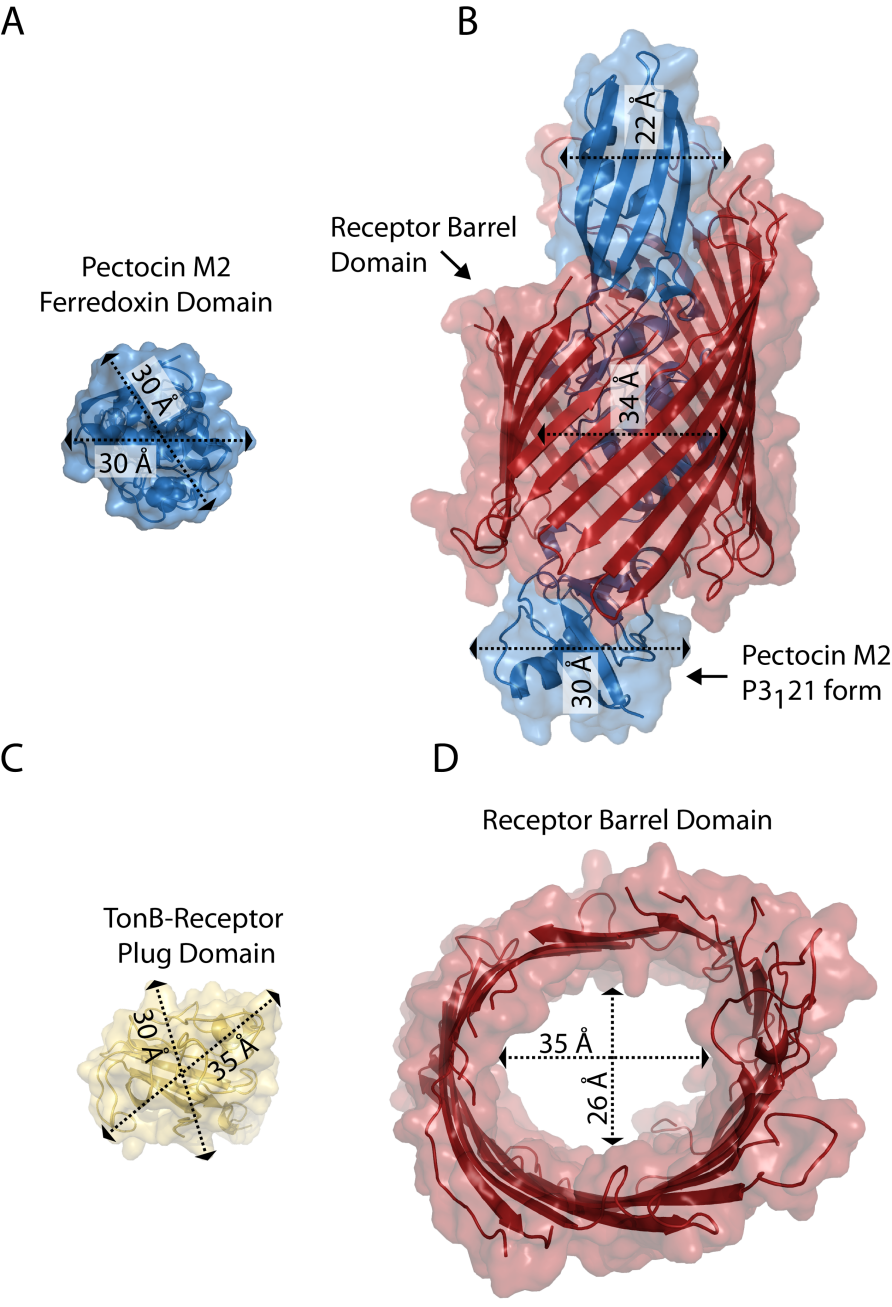
The extended conformation of pectocin M2 has dimensions compatible with passage through the lumen of a TonB-dependent receptor.A. The extended conformation of pectocin M2 (blue cartoon and surface), fitted into the pore of the barrel of HasR from *Serratia marcescens* PDB ID = 3CLS, illustrating that the extended conformation of pectocin M2 is conducive to transport through the lumen of a TonB-dependent receptor without unfolding.B. Dimensions of the ferredoxin domain of pectocin M2.C. The width of the plug domain, which ordinarily blocks the lumen of the receptor barrel, is similar to that of the elongated conformation of pectocin M2.D. Top-down view of the HasR barrel showing the internal dimensions of the barrel domain.

Since pectocin M1 and M2 lack an IUTD they are unable make direct contact with the Tol or Ton complexes in the periplasm and thus are unable to directly utilize the pmf for cell entry. However, the fact that these proteins parasitize a system for which the receptor binding and translocation domains are structurally analogous to the substrate provides an intuitive solution to this problem. A number of TonB-dependent receptors have been identified, which obtain iron from host proteins during infection. In all of these systems the iron or iron containing compound is liberated from the protein on the cell surface and transported into the cell, potentially because all of the proteins identified are too large pass through the lumen of their receptor (Wandersman and Stojiljkovic, 2000; Faraldo-Gomez and Sansom, 2003; Noinaj *et al*., 2012). Plant ferredoxin, however, is a small globular protein, which is in fact comparable in dimensions to the plug domain that ordinarily occludes the pore of a TonB-dependent receptor (Fig. 7A and C). This creates the possibility that the ferredoxin is imported intact into the periplasm. If this were the case it could readily explain the pectocins lack of an IUTD as the energy required for cell entry would still be provided by the Ton complex, but transduced through receptor plug domain, as with ordinary substrate importation.

As the elongated conformation of pectocin M2 has comparable dimensions along its length (Fig. 5A) we manually docked this model into the barrel domain of the hemophore receptor HasR, a TonB-dependent receptor shown to be responsible for importation of the relatively bulky substrate, haem (Fig. 7B and D). This docking illustrates that pectocin M2 in its elongated conformation could traverse the lumen of such a receptor to gain entry to the cell, with the flexibility of pectocin M2 observed in solution allowing the protein to adapt to the shape of the lumen of its transporter during importation. In the case of the transport of iron-siderophores, it is generally thought that there are two possibilities with respect to the role of TonB in stimulating substrate transport. Either TonB induces a rearrangement of the plug domain within the barrel that is sufficient to allow passage of the substrate or it directly pulls the plug domain completely from the barrel (Usher *et al*., 2001; Udho *et al*., 2009; 2012). However, for an intact protein such as ferredoxin to be translocated directly through the lumen of a TonB-dependent receptor, it would be necessary for the plug domain to completely exit the barrel during substrate transport and it has not as yet been directly demonstrated that this occurs in TonB-dependent receptors. The identification of this receptor and the testing of this importation hypothesis represents an intriguing question for future work.

## Experimental procedures

### Expression and purification of ferredoxins and pectocins

A list of bacterial strains and plasmids used in this work is provided in Table S1. The open reading frame for pectocin P minus stop codon was synthesized by DNA 2.0 and ligated into the expression vector pJ404 (T5 promoter, C-terminal His_6_-tag). The resulting vector was designated pJPP1. Vectors for expression of pectocins M1 and M2 were as previously described (Grinter *et al*., 2012a). All proteins were expressed and purified as previously described (Grinter *et al*., 2012a). Proteins were concentrated to 5–15 mg ml^−1^ using a centrifugal concentrator and stored at −80°C in this buffer until required. For pectocins M1 and P, 5% glycerol was added to all buffers as it was found to enhance protein stability.

### Cytotoxicity assays

The cytotoxicity of purified pectocins was tested using the soft agar overlay method (Fyfe *et al*., 1984). 200 μl of mid-log phase culture of the test strain was added to 6 ml of 0.6% agar melted and cooled to 42°C. The molten agar was then overlaid onto LB medium with or without 100–400 μM 2,2′-biyridine. Purified pectocins (2 mg ml^−1^) were spotted directly onto the surface of the overlay, once solidified. Plates were incubated at 28°C for 16 h, and monitored for zones of growth inhibition.

### SAXS data collection and analysis

SAXS data were collected on the X33 beamline at the Deutsches Elektronen Synchrotron (DESY, Hamburg, Germany). Pectocin M2 and pyocin M concentrations between 0.4 and 4.0 mg ml^−1^ were used. Average buffer scattering was subtracted from the sample scattering. The first 200 points (low angle data) of the scattering curve obtained for 1 mg ml^−1^ protein were merged with the rest of the high angle data from the 4 mg ml^−1^ sample to avoid the influence on the data of any inter-particle interference. All data processing was performed using PRIMUS (Konarev *et al*., 2003). Porod-Debye [*I*(q)q^4^ versus q^4^] and normalized Kratky [*I*(q)q^2^ versus q] plots were used to assess particle flexibility as described in the *Results* section (Durand *et al*., 2010; Rambo and Tainer, 2011). The distance distribution function, p(r), was obtained by indirect Fourier transform of the scattering intensity using GNOM (Svergun, 1992). A Guinier plot [ln *I*(s) versus *q*^2^] was used to determine the radius of gyration, *R*_g_, of pectocin M2 and pyocin M. US-SOMO (Rai *et al*., 2005) was used to determine hydrodynamic parameters based on the crystal structures of pectocin M2. CRYSOL (Svergun *et al*., 1995) was used to compute theoretical scattering curves from high-resolution X-ray structures.

### DMD and EOM simulations

Discrete molecular dynamics simulations of the pectocin M2 linker region (residues 96–116) were undertaken with US-SOMO (Brookes *et al*., 2010; Shirvanyants *et al*., 2012) in order to explore pectocin M2 conformational space. The Andersen thermostat temperature (*T*) was set to 0.5 kcal mol^−1^ K^−1^ to allow for sufficient sampling of conformational dynamics around the native state without melting the structure of the linker. The run time and pdb output step were adjusted in order to generate 5000 models. Next, the pool of 5000 ‘random’ pectocin M2 conformers generated was refined by a genetic algorithm implemented in the program GAJOE as part of the ensemble optimization method (Bernadó *et al*., 2007; Petoukhov *et al*., 2012).

### Pectocin M2 crystallization and diffraction data collection

Initial crystallization trials were performed at the high throughput crystallization facility of the University of Zurich using the vapour diffusion method (reservoir volume of 50 μl, drop size: 100 nl protein, 100 nl reservoir solution) with pectocin M2 at a final concentration of 15 mg ml^−1^. Pectocin M2 formed crystals or spherulites in a number of conditions containing ammonium sulphate and PEG 3350. Crystals were extracted from one of these conditions [15% PEG 3350, 0.2 M ammonium sulphate, 3% 2-methyl-2,4-pentanediol (MPD), 0.1 M bis-tris pH 6.5], cryoprotected by increasing PEG 3350 to 30% and data were collected at 100 K to 2.3 Å in the space group *P*2_1_, at the SLS (Zurich). Re-crystallization screening of pectocin M2 was performed, using a custom screen with variations in the concentration/ratio of precipitants from the original condition (ammonium sulphate and PEG 3350), pH and additives. Clusters of large rod-shaped crystals formed at high ammonium sulphate concentrations. This was optimized giving a final condition of 1.8 M ammonium sulphate, 3% MPD, 0.1 M MES, pH 6.5. These crystals were manually separated and cryoprotected with 15–20% glycerol. Data were collected at 100 K on beamlines I02 and I03 at the Diamond Light Source (Oxfordshire, UK). Automatic data-processing was performed with Xia2 within the EDNA package (Incardona *et al*., 2009). Datasets for experimental phasing using the iron–sulphur cluster of pectocin M2 were collected at the iron K-edge (1.7433 Å) and high-resolution data were collected at 0.9796 Å. Data collection statistics from both crystal forms are reported in Table 1.

**Table 1:** Crystallographic data collection and refinement statistics

		High resolution dataset	Iron edge dataset
**Data collection**^a^			
Space group	*P*2_1_	*P*3_1_21	*P*3_1_21
Cell dimensions			
*a*, *b*, *c* (Å)	44.65, 116.75, 60.78	117.45, 117.45, 128.45	117.26, 117.26, 128.53
*α*, *β*, *γ* (°)	90, 94.96, 90	90, 90, 120	90, 90, 120
Resolution (Å)	50.00–2.30 (2.44–2.30)	64.22–1.86 (1.91–1.86)	43.31–2.01 (2.06–2.01)
*R*_merge_ (%)	5.0 (59.9)	3.4 (68.3)	3.8 (69.3)
*R*_pim_ (%)^b^	–	0.9 (15.7)	2.0 (31.0)
Mean *I*/sigma(*I*)	12.41 (1.85)	47.1 (5.5)	28.9 (3.1)
Completeness (%)	96.6 (95.7)	100.0 (99.9)	99.4 (98.7)
Redundancy	3.2 (2.8)	20 (20.8)	8 (6.6)
**Refinement**			
Resolution (Å)	50.00–2.30 (2.44–2.30)	64.22–1.86 (1.91–1.86)	
No. of reflections	52652 (8438)	86092 (6304)	
*R*_work_/*R*_free_ (%)	21.0/27.2	16.8/19.1	
No. of atoms			
Protein	4093	4175	
Ligand/ion	34	388	
Water	49	438	
*B* factors			
Protein	69.1	45.5	
2Fe-2S	74.6	35.4	
SO4^2−^/Cl^−^	95	68.2	
Glycerol/MPD	–	70.7	
Water	42.4	56.0	
Root mean square deviations			
Bond lengths (Å)	0.016	0.024	
Bond angles (°)	1.757	2.55	
PDB identifier	4N59	4N58	

a Values in parentheses refer to the highest resolution shell.

b *R*_pim_ = *Σ*_hkl_[1/(*N* − 1)^1/2^*Σ*_i_|*I*_i_(hkl) − < *I*(hkl) > |/*Σ*_hkl_*Σ*_i_*I*_i_(hkl)

### Pectocin M2 structure solution and refinement

Phases for the *P*2_1_ and *P*3_1_21 datasets were obtained from the anomalously scattering substructure from the pectocin–ferredoxin domain iron–sulphur cluster, determined for the iron-edge dataset using the Hybrid Substructure Search from the Phenix package (Adams *et al*., 2010). Four positions were located per ASU corresponding to two iron–sulphur clusters (correlation coefficient = 0.5) from two pectocin M2 molecules. These positions were then utilized by Phaser-EP (McCoy *et al*., 2007; Adams *et al*., 2010; Winn *et al*., 2011) phases were improved using RESOLVE density modification from the Phenix package, and the initial model was built and refined using Phenix Autobuild (Adams *et al*., 2010). The model was then built and refined manually using Coot 0.7 and Refmac5 (McCoy *et al*., 2007; Emsley *et al*., 2010; Murshudov *et al*., 2011). Validation of refined structures was performed using the Molprobity web server and Procheck from CCP4i (Laskowski *et al*., 1993; Chen *et al*., 2010). Refinement statistics for both datasets are reported in Table 1.

### Lipid II cleavage assay

Lipid II hydrolysis assays were performed and visualized as previously described by (Grinter *et al*., 2012b), with non-radiolabelled lipid II substrate obtained from the UK Bacterial Cell Wall Biosynthesis Network (Lloyd *et al*., 2008; Clarke *et al*., 2009). A band corresponding to lipid II was observed with an R_f_ of 0.7 as reported previously (Barreteau *et al*., 2009).
