# Supplementary material for: Structure of the atypical bacteriocin pectocin M2 implies a novel mechanism of protein uptake
Source: Mol Microbiol. 2014 Jun 18;93(2):234–46. doi: 10.1111/mmi.12655 (PMC4671253; doi:10.1111/mmi.12655)
Supplement: Supplementary file 1 [file mmi0093-0234-sd1.pdf]

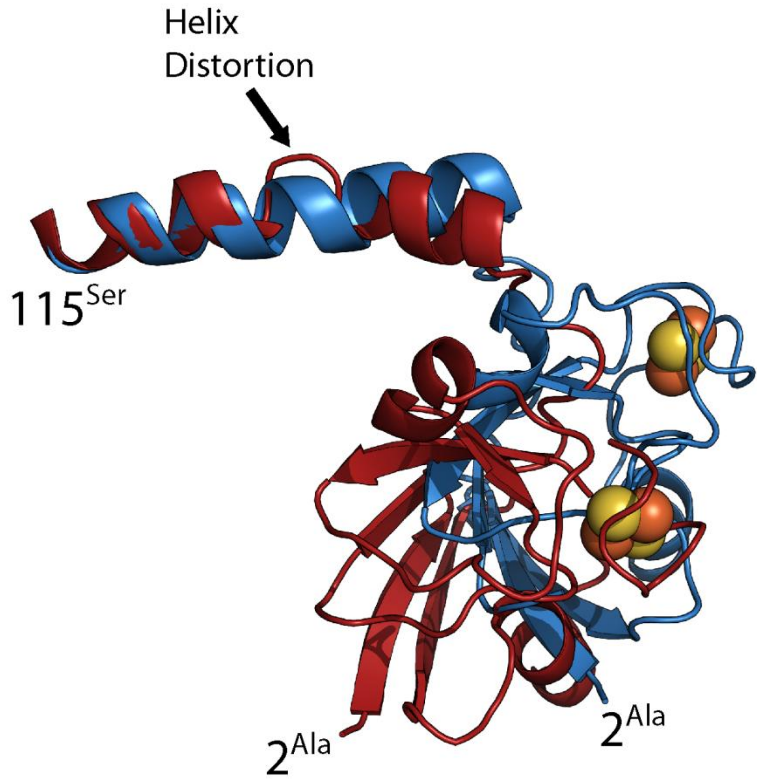

Figure S1. Difference in orientation of monomers of pectocin M2 in the ASU of the P2$_1$ form. When aligned based on the catalytic domain (AA 116-271) the ferredoxin domains of the molecules in the P2$_1$ ASU differ significantly in orientation, due to distortion of the linker helix.

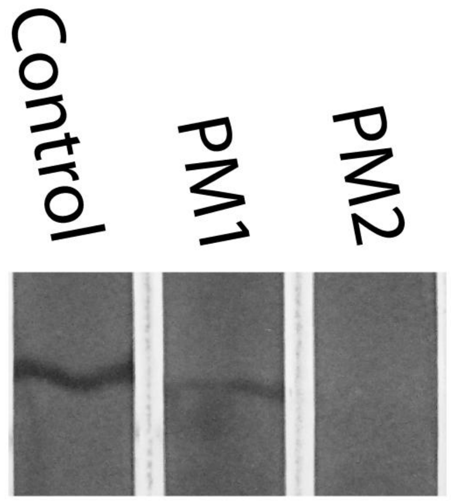

Figure S2. Pectocin M1 and M2 cleave lipid II. Thin layer chromatography plate showing degradation of lipid II after incubation with either pectocin M1 (PM1) or pectocin M2 (PM2). Control reaction contained no protein. The observed band corresponding to lipid II migrated at the expected Rf of 0.7, compared to the solvent front.

Table S1. Bacterial strains and plasmids utilised in this study, and Pectobacterium susceptibility to pectocins

Strain or Plasmid	Relevant Characterisitc(s)	Suceptible to Pectocin			Source or Reference
		M1	M2	P	
E. coli					
DH5α	*F^-, φ80dlacZΔM15, Δ(lacZYA-argF)U169, deoR, recA1, endA1, hsdR17(rk^-$, mk^+$), phoA, supE44, λ^-, thi-1, gyrA96, relA1*				Invitrogen
BL21D3	F– *omp*T *hsd*SB(rB–, mB–) *gal dcm* (DE3)				Invitrogen
P. carotovorum subsp. carotovorum					
LMG 2378	Isolated from Solanum tuberosum	*	*	*	BCCM
LMG 2383	Isolated from Solanum tuberosum	*	*	*	BCCM
LMG 2384	Isolated from Solanum tuberosum	*	*	*	BCCM
LMG 2385	Isolated from Solanum tuberosum	*	*	*	BCCM
LMG 2390		*	*	*	BCCM
LMG 2394	Isolated from Solanum tuberosum	*	*	*	BCCM
LMG 2410	Isolated from *Cucumis sativus*				BCCM
LMG 2412	Isolated from *Hyacinthus orientalis*	*	*	*	BCCM
LMG 2442	Isolated from *Brassica oleracea*				BCCM
LMG 2444	Isolated from *Solanum tuberosum* (tuber soft rot)				BCCM
LMG 2913	Isolated from soil	*		*	BCCM
P. atrosepticum					
LMG 2374	Isolated from Apium graveolens var. dulce	*	*	*	BCCM
LMG 2375	Isolated from *Solanum tuberosum* (tuber soft rot)	*	*	*	BCCM
LMG 2386	Isolated from *Solanum tuberosum* (stem rot)	*	*	*	BCCM
LMG 2391	Isolated from soil	*		*	BCCM
LMG 2413	Isolated from soil	*	*	*	BCCM
LMG 2443	Isolated from *Brassica oleracea*	*	*	*	BCCM
LMG 2454	Isolated from *Chrysanthemum morifolium*	*	*	*	BCCM
SCRI 1043	Isolated from *Solanum tuberosum* (tuber soft rot)	*		*	SCRI
Plasmid					
pJexpress404	Amp^{r,} cloning/expression vector, T5 promoter				DNA 2.0
pET21-a(+)	Amp^{r,} cloning/expression vector, T7 promoter				Novagen
pETMPCI	Amp^{r,} pET21-a(+) with Pectocin M1 inserted into NdeI/XhoI sites				this study
pETMBPR	Amp^{r,} pET21-a(+) with Pectocin M2 inserted into NdeI/XhoI sites				this study
pJPP1	Amp^{r,} pJexpress404 with Pectocin P inserted into NdeI/XhoI sites				this study
